# Metabolische/bariatrische Chirurgie ist effektiv: Was ist die Evidenz zu harten Endpunkten und zum Langzeitverlauf?

**DOI:** 10.1007/s00104-025-02392-y

**Published:** 2025-10-22

**Authors:** Daniel Moritz Felsenreich, Gerhard Prager

**Affiliations:** Universitätsklinik für Allgemeinchirurgie, Klinische Abteilung für Viszeralchirurgie, Medizinische Universität Wien, Währinger Gürtel 18–20, 1090 Wien, Österreich

**Keywords:** Sleeve gastrectomy, Single anastomosis duodeno-ileal bypass mit Sleeve gastrectomy, Langzeitoutcome, Y‑Roux-Magenbypass, Nachsorge, Sleeve gastrectomy, Single anastomosis duodeno-ileal bypass with Sleeve gastrectomy, Long-term outcome, Roux-en‑Y gastric bypass, Aftercare

## Abstract

Metabolische/bariatrische Chirurgie gilt als sichere und effektivste Therapie bei Adipositas und ihren Begleiterkrankungen. Sie führt nicht nur zu nachhaltigem Gewichtsverlust, sondern verbessert auch Stoffwechselstörungen wie Typ-2-Diabetes, senkt das Risiko für bestimmte Krebserkrankungen und erhöht die Lebenserwartung. Weltweit am häufigsten durchgeführt werden die „Sleeve gastrectomy“ (Schlauchmagen), der Y‑Roux Magenbypass und der „one anastomosis bypass“. Neue Operationen wie der „Single anastomosis duodeno-ileal bypass“ + „Sleeve gastrectomy“ (SADI-S) zeigen vielversprechende Ergebnisse, insbesondere bei Patient:innen mit hohem Body Mass Index (BMI). Mittlerweile gibt es eine Vielzahl an Studien mit harten Endpunkten und Langzeitoutcomes. Die Wahl der Operationsmethode erfolgt individuell und interdisziplinär. Entscheidend für den langfristigen Erfolg ist eine lebenslange Nachsorge. Zahlreiche Studien belegen darüber hinaus eine deutliche Verbesserung der Lebensqualität sowie eine Reduktion der Gesamtmortalität durch metabolische/bariatrische Chirurgie.

## Abstract (en)

Metabolic/bariatric surgery is considered to be safe and the most effective treatment for obesity and its associated medical problems. It not only leads to sustained weight loss but also improves a variety of metabolic disorders, such as type 2 diabetes, reduces the risk of several obesity-associated cancers and increases life expectancy. The most commonly performed procedures worldwide are Sleeve gastrectomy, Roux-en‑Y gastric bypass and one anastomosis gastric bypass. Newer techniques, such as single anastomosis duodeno-ileal bypass with sleeve gastrectomy (SADI-S) show promising results, especially in patients with a high body mass index (BMI). A growing number of studies now provide data on hard clinical endpoints and long-term outcomes. The choice of surgical procedure is made individually and within an interdisciplinary setting. Lifelong follow-up care is crucial to ensure long-term success. Numerous studies also demonstrate significant improvements in the quality of life and a reduction in overall mortality through metabolic/bariatric surgery.

Im folgenden Text werden die Wirkungsmechanismen, die Indikation und der weltweite Stellenwert von metabolischen/bariatrischen Operationen erläutert. Die heute wichtigsten Operationen werden erklärt und vorhandene Studien zu Langzeitdaten (Gewichtsverlust, Outcome von Begleiterkrankungen und unerwünschte Wirkungen) beschrieben. Des Weiteren werden die bedeutenden Outcomes des Malignomrisikos nach metabolischer/bariatrischer Chirurgie, der Lebenserwartung und Lebensqualität gesondert beschrieben und anhand großer Studien eingeordnet.

Adipositas stellt eine chronische Erkrankung dar, die sowohl die Lebensqualität als auch die Lebenserwartung erheblich beeinträchtigen kann. Besonders relevant sind in diesem Zusammenhang die Begleiterkrankungen des metabolischen Syndroms, darunter Typ-2-Diabetes mellitus (T2DM), Bluthochdruck, obstruktive Schlafapnoe, Fettstoffwechselstörungen, „metabolic dysfunction-associated steatotic liver disease“ (MAFLD) und ihre entzündliche Form, die „metabolic dysfunction-associated steatohepatitis“ (MASH). Zusätzlich belastet Adipositas das muskuloskeletale System erheblich [1].

Mit zunehmendem Körpergewicht steigt außerdem das Risiko für die Entwicklung bestimmter Krebsarten [2] sowie für das Auftreten mikro- und makrovaskulärer Komplikationen wie Herzinfarkt oder Schlaganfall [3, 4]. Seit 1975 hat sich die weltweite Prävalenz von Adipositas fast vervierfacht: Aktuell gelten über 2,5 Mrd. erwachsene Menschen als übergewichtig, davon sind etwa 890 Mio. adipös [5]. Epidemiologische Daten zeigen, dass die niedrigste Mortalität im Bereich eines Body Mass Index (BMI) von 18,5–25,0 kg/m^2^ liegt. Mit jeder Zunahme des BMI um 5 kg/m^2^ steigt die Sterblichkeit um etwa 30 % [6].

Gegenwärtig gilt die metabolische/bariatrische Chirurgie (MBC) als die effektivste und nachhaltigste Behandlungsoption für Menschen mit Adipositas. Neben der Reduktion des Körpergewichts können durch die operativen Eingriffe auch die bestehenden Begleiterkrankungen verbessert oder vollständig zurückgebildet/in Remission geführt werden. Darüber hinaus lässt sich das Risiko für die Entwicklung neuer metabolischer Erkrankungen deutlich senken [7].

## Wirkungsmechanismus von metabolischer/bariatrischer Chirurgie

Aus anatomisch-funktioneller Sicht wirken metabolische/bariatrische Eingriffe über unterschiedliche Mechanismen: einerseits durch restriktive Maßnahmen wie die Verkleinerung des Magenvolumens, wodurch die Nahrungsaufnahme reduziert wird, und andererseits durch hypoabsorptive Komponenten, bei denen durch eine Verkürzung des resorptiven Dünndarmabschnitts die Nährstoffaufnahme eingeschränkt wird. Viele Verfahren kombinieren beide Ansätze.

Darüber hinaus beeinflusst MBC die hormonelle Regulation des Appetits: So kommt es beispielsweise nach einem Magenbypass zu einer verstärkten Ausschüttung von „glucagon-like peptid 1“ (GLP-1) und Peptid YY, was zu einem früheren Sättigungsgefühl führt [8]. Nach einer „Sleeve gastrectomy“ (SG) wird die Produktion des appetitanregenden Hormons Ghrelin signifikant reduziert [8]. Auch die Insulinsensitivität verschiedener Gewebe verbessert sich, was maßgeblich zur Remission metabolischer Begleiterkrankungen beiträgt [9].

Weitere bedeutende Veränderungen betreffen das intestinale Mikrobiom sowie die Zusammensetzung und Funktion der Gallensäuren, die beide nach MBC moduliert werden [8].

## Indikation und Kontraindikationen zur metabolischen/bariatrischen Chirurgie

Rezent wurden modernisierte, evidenzbasierte Leitlinien zur Indikationsstellung für metabolische/bariatrische Operationen von der International Federation for the Surgery of Obesity and Metabolic Disorders (IFSO) in Kooperation mit der American Society for Metabolic and Bariatric Surgery (ASMBS) veröffentlicht [10, 11].

Demnach kann ein operatives Verfahren zur Gewichtsreduktion bereits ab einem BMI von ≥ 35 kg/m^2^ empfohlen werden – unabhängig davon, ob Begleiterkrankungen vorliegen oder nicht. Bei Patient:innen mit einem BMI zwischen 30 und 35 kg/m^2^ sollte ein chirurgischer Eingriff erwogen werden, sofern sich durch konservative Maßnahmen weder ein stabiler Gewichtsverlust noch eine signifikante Verbesserung bestehender assoziierter Erkrankungen erzielen lassen [10, 11]. Diese Leistungen werden derzeit allerdings noch nicht von allen Krankenkassen refundiert.

Ein operatives Verfahren zur Gewichtsreduktion kann ab einem BMI von ≥ 35 kg/m^2^ empfohlen werden

Eine feste Altersobergrenze für die Durchführung von MBC besteht laut aktuellen Empfehlungen nicht. Allerdings ist eine umfassende präoperative Abklärung bestehender Vorerkrankungen essenziell. Zudem kann chirurgische Adipositastherapie bei ausgeprägter Adipositas auch präventiv/als Überbrückung („bridging“) wirken und das Risiko sowie die Komplikationsrate bei anderen chirurgischen Eingriffen – etwa in der Hernien‑, Transplantations- oder Gelenkchirurgie – deutlich reduzieren [10].

Die Betreuung betroffener Patient:innen im Rahmen eines interdisziplinären Teams trägt wesentlich zur Minimierung perioperativer Komplikationen und zur Verbesserung des Outcomes bei. Die abschließende Indikationsstellung für den operativen Eingriff sollte jedoch in der Verantwortung des/der behandelnden Chirurg:in liegen. Da es sich bei Adipositas um eine chronische Erkrankung handelt, kann es im Langzeitverlauf notwendig sein, ergänzende Maßnahmen wie adjuvante Therapien (medikamentös, endoskopisch etc.) oder Revisionsoperationen durchzuführen, um den gewünschten Therapieerfolg zu sichern [10].

Für Patient:innen mit einem BMI ≥ 50 kg/m^2^ gelten konservative Therapieansätze zur Gewichtsreduktion als weitgehend ineffektiv/nicht ausreichend. In diesen Fällen sollte eine bariatrische Operation zeitnah und ohne unnötige Verzögerung erfolgen. Andere Patient:innengruppen hingegen sollten vor einer chirurgischen Maßnahme zunächst konservative Strategien zur Gewichtsreduktion durchlaufen haben [10].

Als (relative) Kontraindikationen für bariatrische Eingriffe gelten unter anderem eine mangelnde Bereitschaft zur lebenslangen Einnahme von Vitaminpräparaten und zur regelmäßigen Nachsorge sowie bestehender Alkohol- oder Drogenmissbrauch. Auch bestimmte psychiatrische Erkrankungen können einer Operation entgegenstehen [12].

## Metabolische/bariatrische Operationsmethoden

Weltweit werden jährlich über 480.000 metabolische/bariatrische Eingriffe vorgenommen [13]. Dabei stellt die „Sleeve gastrectomy“ (SG, Schlauchmagen) mit über 60 % aller Operationen den am häufigsten durchgeführten Eingriff dar. An zweiter Stelle folgt der Y‑Roux-Magenbypass (RYGB) mit etwa 30 %, gefolgt vom „one anastomosis gastric bypass“ (OAGB, Omega-Loop-Magenbypass) mit nur deutlich geringeren 4,3 % [13]. Eine neuere Operationsmethode stellt der von der IFSO bereits anerkannte SADI‑S („Single anastomosis duodeno-ileal bypass + Sleeve gastrectomy“) dar, der sowohl in Europa als auch in den USA zunehmend an Bedeutung gewinnt.

In Europa werden jährlich etwa 180.000 MBC durchgeführt, wobei ebenfalls die SG vor dem RYGB die häufigste Operation ist, allerdings mit deutlich geringerem Abstand. Auch OAGB wird in Europa mit 16,0 % häufiger durchgeführt [14].

Andere Verfahren wie das Magenband, „gastric plication“ (Magenfaltung) oder die biliopankreatische Diversion (BPD) spielen heute eine deutlich untergeordnete Rolle und finden sowohl in Europa als auch weltweit nur noch selten Anwendung [13, 14].

Die „Sleeve gastrectomy“ stellt den am häufigsten durchgeführten Eingriff weltweit dar

Zunehmend an Bedeutung gewinnen auch endoskopische Verfahren (z. B. „endoscopic sleeve gastroplasty“ [ESG]) und medikamentöse Therapien zur Gewichtsreduktion [15, 16]. Diese Verfahren/Therapien können auch sekundär (z. B. „transoral outlet reduction“ [TORe]) nach vorangegangener Adipositaschirurgie eingesetzt werden. Allerdings gibt es bisher wenige Daten bezüglich des Langzeittherapieerfolgs. Des Weiteren können auch bei diesen Verfahren/Therapien Komplikationen auftreten [15, 16].

## Langzeitdaten nach Operationsmethoden

### „Sleeve gastrectomy“ (Schlauchmagen)

**Abb. 1:**
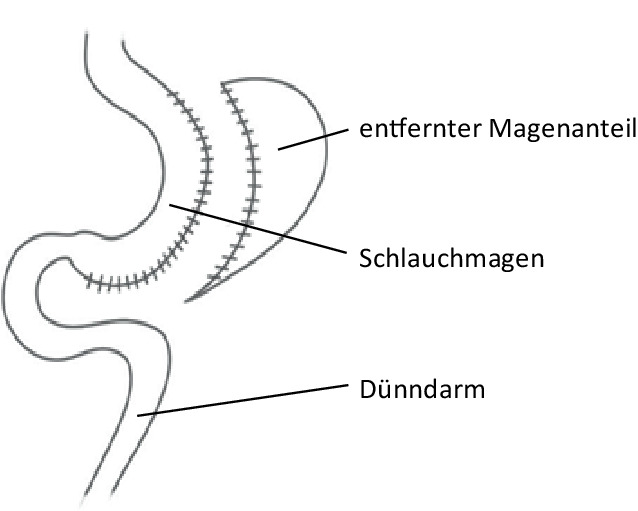
Schlauchmagen („sleeve gastrectomy“). (Aus [42], mit freundl. Genehmigung, © D.M. Felsenreich, G. Prager, CC BY 4.0, https://creativecommons.org/licenses/by/4.0/)

Bei der SG (Abb. 1) werden mittels eines Staplers etwa 80–85 % des Magens entlang der großen Kurvatur entfernt. Übrig bleibt ein schlauchförmiger Restmagen. Ein entscheidender Aspekt dieses Verfahrens ist die vollständige Resektion des dehnbaren Fundusbereichs. Da weder der Pylorus noch der Dünndarm bei dieser Operation verändert werden, bleibt die Möglichkeit einer endoskopischen retrograden Cholangiopankreatikographie (ERCP) auch postoperativ bestehen [17].

Langfristig können nach SG jedoch bestimmte Nachteile auftreten. Dazu zählen insbesondere eine erneute Gewichtszunahme („recurrent weight gain“) sowie ein Wiederauftreten von bereits verbesserten Begleiterkrankungen. Des Weiteren kann durch die SG die Entwicklung oder Verschlechterung eines präoperativ bestehenden gastroösophagealen Refluxes (GERD) begünstigt werden.

In einer Studie zur SG mit einem minimalen Follow-up von 15 Jahren konnte gezeigt werden, dass nach diesem Zeitraum bereits 50 % der Patient:innen wegen GERD oder Gewichtszunahme auf eine andere Methode (meist Long-limb-RYGB) konvertiert werden mussten. Trotz dieser bereits hohen Konversionsrate wurden bei einigen der nicht konvertierten Patient:innen Refluxzeichen in der Gastroskopie (Ösophagitis, Barrett-Metaplasie) und/oder ein erhöhter De-Meester-Score in der 24-h-pH-Metrie diagnostiziert [18]. Ähnliches zeigt sich bei der SM-BOSS-Studie, einer randomisiert kontrollierten Studie, die SG mit RYGB bei einem Follow-up von über 10 Jahren vergleicht: Es konnte gezeigt werden, dass der RYGB sowohl bei Gewichtverlust, Konversionsrate als auch GERD-Entwicklung der SG überlegen ist [19]. In einem rezenten systematischen Review von Brown zeigt sich die SG im Vergleich zum RYGB im Mittel- bis Langzeit-Follow-up prinzipiell effektiv bezüglich Gewichtsverlust und Verbesserung von Begleiterkrankungen bei sogar etwas besserem Sicherheitsprofil zugunsten der SG [20]. In der SLEEVEPASS-Studie, die ebenfalls RYGB mit SG randomisiert kontrolliert vergleicht, konnten ähnliche Ergebnisse erzielt werden. Dies wird im nächsten Abschnitt über Y‑Roux Magenbypass (Abschn. „Y-Roux Magenbypass“) noch genauer beschrieben [21, 22].

Zusammenfassend sollte in jedem Fall die Indikation zur SG aufgrund der unerwünschten Wirkungen mit Bedacht gestellt werden.

### Y-Roux-Magenbypass

**Abb. 2:**
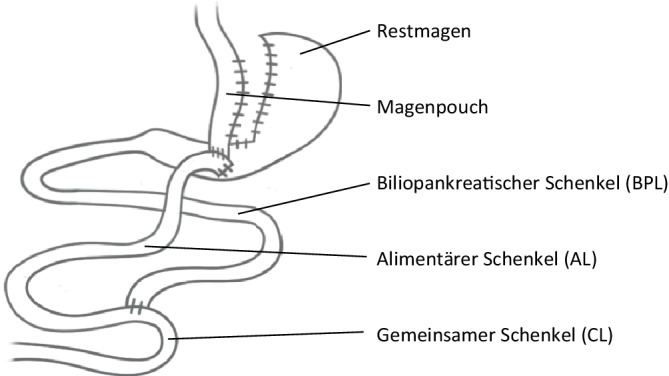
Y‑Roux-Magenbypass. (Aus [42], mit freundl. Genehmigung, © D.M. Felsenreich, G. Prager, CC BY 4.0, https://creativecommons.org/licenses/by/4.0/)

Beim RYGB (Abb. 2) wird einerseits eine kleine Magentasche („Pouch“) geformt, und andererseits entstehen durch Anlegen einer Gastrojejunostomie und einer Jejunojejunostomie ein biliopankreatischer Schenkel (BPL) und ein alimentärer Schenkel (AL). In der Literatur werden unterschiedliche Pouchformen und Längen der beiden Schenkel angegeben, wobei die Länge des BPL den meisten Einfluss auf den Gewichtsverlust hat. Die gemeinsame Endstrecke (CL) ist meist variabel in der Länge [23].

Der RYGB zählt zu den am besten untersuchten MBC mit umfassenden Langzeitdaten. So zeigt beispielsweise die Swedish Obesity Study (SOS), dass ein signifikanter Gewichtsverlust auch noch bis zu 26 Jahre nach dem Eingriff erhalten werden kann. Besonders die RYGB-Gruppe weist im Vergleich zur Magenbandgruppe eine deutlich geringere Konversionsrate auf [24]. In der finnischen SLEEVEPASS Study, einer randomisiert kontrollierten Studie, die RYGB mit SG vergleicht, zeigt sich nach 10 Jahren ebenfalls ein besserer Gewichtsverlust in der RYGB-Gruppe bei geringerer Ösophagitisrate (im Vergleich zur SG-Gruppe) in der Gastroskopie. Bezüglich Vitaminstatus gab es keinen Unterschied in den beiden Gruppen, wobei Mängel in beiden Gruppen gering waren [21, 22]. Eine aktuelle Metaanalyse, die bei über 600 Patient:innen (aus randomisiert kontrollierten Studien) RYGB mit konservativer Diabetestherapie verglichen hat, konnte eine signifikant bessere Performance (höhere Remissionsrate) in der chirurgischen Gruppe darstellen [25]. Vergleichende Funktionsdiagnostikstudien, die RYGB mit SG bezüglich Säurebelastung des Ösophagus untersuchten, zeigten eine signifikant höhere Refluxrate nach SG verglichen mit RYGB [25].

Zusammenfassend ist der RYGB eine MBC mit hervorragenden Langzeitdaten bezüglich Gewichtsverlust und Remission von Begleiterkrankungen bei niedriger Revisionsrate und niedriger Refluxwahrscheinlichkeit.

### „One anastomosis gastric bypass“ (Omega-Loop-Magenbypass)

**Abb. 3:**
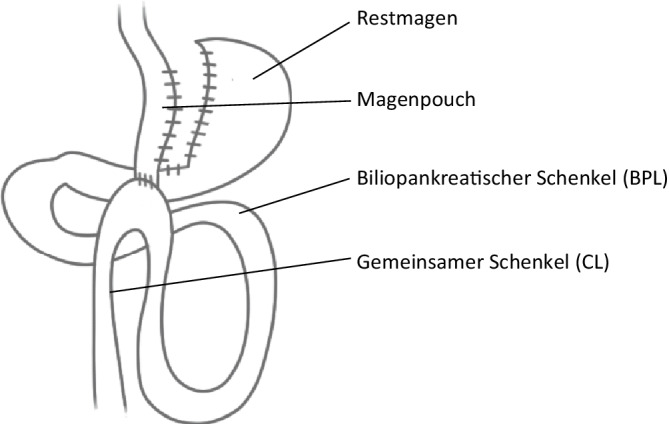
„One anastomosis gastric bypass“ (Omega-Loop Magenbypass). (Aus [42], mit freundl. Genehmigung, © D.M. Felsenreich, G. Prager, CC BY 4.0, https://creativecommons.org/licenses/by/4.0/)

Beim OAGB (Abb. 3) wird im Gegensatz zum RYGB nach der Formung des Pouches (der in der Regel deutlich länger und sehr schlank ausgeführt wird) nur eine Gastrojejunostomie gemacht und auf die Erstellung einer Fußpunktanastomose verzichtet [26]. Zu den Vorteilen dieses Verfahrens zählen eine verkürzte Operationszeit sowie ein insgesamt reduziertes Risiko für das Auftreten innerer Hernien, ein möglicher Nachteil besteht jedoch in der Entwicklung eines biliären Refluxes in den Pouch oder sogar in den Ösophagus [27]. Dieses Problem zeigt sich bei etwa 4–5 % der Patient:innen im Langzeitverlauf, lässt sich jedoch durch eine Konversion in einen RYGB einfach beheben [28]. Eine randomisiert kontrollierte Studie, die SG mit dem OAGB in einem 7‑Jahres-Follow-up vergleicht, zeigt einen signifikant besseren Gewichtsverlust sowie eine höhere Remissionsrate von Begleiterkrankungen in der OAGB-Gruppe. Auch das Outcome, gemessen mit dem BAROS-Score, ist bei den OAGB-Patient:innen signifikant besser [29]. In einer OAGB-Langzeit-Follow-up-Studie mit 1917 Patient:innen konnten nach 9 Jahren noch immer über 30 % „total body weight loss“ (TWL%) gemessen werden. Auch die Remissionsraten der Begleiterkrankungen waren nach diesem langen Zeitraum noch zwischen 55 und 80 % [30]. Im YOMEGA Trial, eine weitere randomisiert kontrollierte multizentrische Studie, die OAGB mit RYGB vergleicht, zeigte sich nach 5 Jahren kein signifikanter Unterschied in den beiden Operationsmethoden bezüglich des sehr guten Gewichtsverlusts und der Remission von Begleiterkrankungen. GERD war allerdings in der OAGB-Gruppe signifikant häufiger als in der RYGB-Gruppe [31].

Insgesamt zeigen sich nach OAGB auch im Langzeitverlauf exzellente Ergebnisse, wobei ein gewisses Risiko der Entwicklung von galligem Reflux besteht, wodurch eine Konversion auf RYGB notwendig werden würde.

### „Single anastomosis duodeno-ileal bypass + Sleeve gastrectomy“

**Abb. 4:**
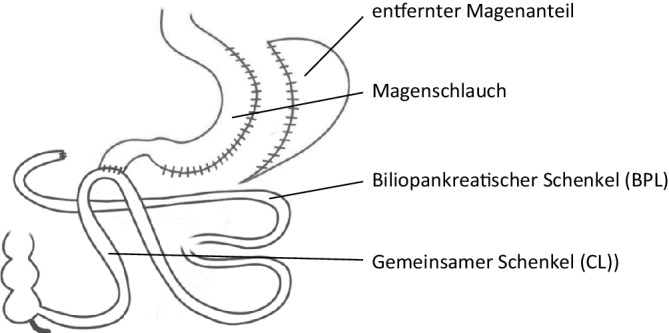
„Single anastomosis duodeno-ileal bypass + sleeve gastrectomy“ (SADI-S). (Aus [42], mit freundl. Genehmigung, © D.M. Felsenreich, G. Prager, CC BY 4.0, https://creativecommons.org/licenses/by/4.0/)

SADI‑S (Abb. 4) wird aktuell vorrangig bei Patient:innen mit einem höheren BMI (über 45 kg/m^2^) eingesetzt und wurde nach Prüfung der Datenlage von der IFSO als sicher und effektiv eingestuft [32]. Die Operation kombiniert eine SG mit einer Duodenoileostomie 3–4 cm nach dem Pylorus. Dabei wird der gesamte in der Essenspassage gelegene Dünndarm (CL) vermessen und standardisiert (250–300 cm) [33].

Langzeitdaten von bis zu 6 Jahren zeigen vielversprechende Resultate: ein durchschnittlicher BMI-Rückgang von mehr als 17 kg/m^2^ sowie eine Diabetesremissionsrate von 77 % [34]. In einer Metaanalyse (8 Studien und 4259 Patient:innen) wurde SADI‑S mit RYGB verglichen. Dabei war SADI‑S sowohl in den Bereichen Gewichtsverlust als auch Diabetesremission und Safety-Profil dem RYGB überlegen.

Insgesamt ist SADI‑S eine MBC mit viel Potenzial in der Zukunft. Vorteile sind ein standardisierter „common channel“ (Länge des Dünndarms, in der Darmpassage) sowie der Pylorus in der Essenspassage (weniger Risiko für Dumping). Ergebnisse randomisiert kontrollierter Studien mit direkten Vergleichen zu anderen Operationsmethoden bleiben aber noch abzuwarten.

## Harte Endpunkte zu Outcomes nach metabolischer/bariatrischer Chirurgie

### Malignomrisiko nach metabolischer/bariatrischer Chirurgie

Bekannterweise sind die mit Adipositas assoziierten Erkrankungen des metabolischen Syndroms mit erhöhtem Risiko der Entwicklung von Malignomen vergesellschaftet. In einer großen Studie von Aminian et al. (SPLENDID) mit über 30.000 inkludierten Patient:innen konnte gezeigt werden, dass durch MBC nicht nur das Risiko, ein Malignom zu entwickeln, signifikant verringert wird, sondern auch die malignomspezifische Mortalität gesenkt wird. Am stärksten ausgeprägt war hierbei die Risikoreduktion des Endometriumkarzinoms gefolgt vom Mammakarzinom [35].

Durch MBC wird das Malignomrisiko signifikant verringert und die malignomspezifische Mortalität gesenkt

Eine Kohortenstudie von Åkerström et al. untersuchte das Risiko für Ösophagusadenokarzinome nach MBC. Besonders relevant ist dies vor dem Hintergrund, dass einige MBC mit einem erhöhten Risiko für GERD einhergehen, was potenziell eine Kaskade von chronischer Schleimhautreizung über Barrett Metaplasie/Dysplasie bis hin zum Entstehen eines Adenokarzinoms im Ösophagus in Gang setzen könnte. Diese Studie mit 748.932 Patient:innen ergab, dass das Risiko für ein Adenokarzinom des Ösophagus nach einem RYGB im Vergleich zur adipösen Allgemeinbevölkerung vermindert war. Interessanterweise nahm das Risiko für ein Adenokarzinom nach RYGB mit zunehmender Dauer des Follow-up sogar weiter ab [36].

Insgesamt konnten diese großen Analysen zeigen, dass das Malignomrisiko nach MBC reduziert ist, wobei der Effekt bei hormonabhängigen Tumoren am höchsten zu sein scheint.

### Lebenserwartung nach metabolischer/bariatrischer Chirurgie

Die Steigerung der durch Adipositas reduzierten Lebenserwartung ist einer der wichtigsten Effekte von MBC. In einer Metaanalyse („matched cohorts“) mit über 1470 inkludierten Artikeln und 174.772 Patient:innen konnte gezeigt werden, dass durch MBC die mittlere Lebenserwartung um 6,3 Jahre gesteigert werden konnte. In der Gruppe von Patient:innen mit Diabetes sogar um 9,3 Jahre [37]. Auch eine weitere Studie von Carlsson mit 2010 Patient:innen konnte durch MBC eine Reduktion der Gesamtmortalität und der kardiovaskulären Mortalität sowie eine Erhöhung der Lebenserwartung feststellen. Dieses Outcome konnte sowohl in der Gruppe mit als auch ohne Diabetes bestätigt werden [38].

Die Daten dieser großen Analysen zeigen deutlich den starken Effekt der MBC, der v. a. der Remission der Begleiterkrankungen geschuldet ist und ein gutes Argument für eine metabolische/bariatrische Operation darstellt.

### Lebensqualität nach metabolischer/bariatrischer Chirurgie

Subjektiv eines der wichtigsten Outcomes für die Patient:innen ist die Verbesserung der Lebensqualität (QOL) nach MBC. Eine aktuelle randomisiert kontrollierte Studie, die bei 628 Patient:innen RYGB und SG bezüglich QOL verglichen hat, konnte zeigen, dass nach beiden Operationen die Langzeitlebensqualität (in verschiedenen Fragebögen) nach über 5 Jahren sehr gut ist. Einzig durch GERD-Symptomatik nach SG ergibt sich ein kleiner Unterschied zugunsten RYGB [39]. In einer weiteren Studie, welche die Lebensqualität über 10 Jahre nach SG ermittelt hat, konnten ebenfalls gute Resultate erzielt werden. Es zeigte sich aber trotzdem, dass GERD und Gewichtswiederzunahme signifikante Auswirkungen auf die Lebensqualität haben können [40]. Eine weitere randomisiert kontrollierte Studie von Simonson et al. zur Lebensqualität nach MBC und konservativer Therapie bei 228 Patient:innen mit Adipositas konnte zeigen, dass MBC sogar über einen Zeitraum von 12 Jahren hinweg überlegen ist. Nicht nur die körperliche Lebensqualität, sondern auch das allgemeine Gesundheitsgefühl, die körperliche Leistungsfähigkeit, und das Energieniveau (Vitalität) waren in der MBC-Gruppe höher als in der konservativen Therapiegruppe [41].

## Schlussfolgerung

Metabolische/bariatrische Chirurgie ist sehr sicher und die derzeit wirksamste Behandlungsoption bei Adipositas und den damit verbundenen Begleiterkrankungen. Besonders bei Patient:innen mit Diabetes mellitus sollte eine operative Therapie frühzeitig in Betracht gezogen werden – auch bei einem niedrigeren BMI –, um eine Remission zu ermöglichen oder ein Fortschreiten der Erkrankung zu vermeiden. Insgesamt sind mittlerweile viele Studien zu Langzeitdaten und harten Endpunkten nach MBC vorhanden, die diesen Operationen eine sehr gute Wirkung bescheinigen. Auf individueller Ebene ist die Auswahl des am besten geeigneten Operationsverfahrens essenziell. Unabhängig vom gewählten Eingriff ist eine lebenslange Nachsorge unerlässlich, um langfristig stabile und erfolgreiche Behandlungsergebnisse sicherzustellen.

## Fazit für die Praxis

- Zahlreiche Studien mit Langzeitverlauf und harten Endpunkten zeigen eine anhaltende Gewichtsreduktion, Verbesserung metabolischer Erkrankungen sowie eine Senkung der Mortalität und eine Steigerung der Lebenserwartung und Lebensqualität nach metabolischer/bariatrischer Chirurgie.
- Das Risiko, an einem Malignom zu erkranken, ist nach metabolischer/bariatrischer Chirurgie reduziert.
- Bei Patient:innen mit Typ-2-Diabetes sollte die Indikation zur metabolisch-bariatrischen Operation frühzeitig gestellt werden, um eine Remission zu fördern und das Fortschreiten der Erkrankung zu vermeiden.
- Die Wahl der Operationsmethode sollte individuell unter Einbeziehung eines interdisziplinären Teams erfolgen.
